# Pathogenesis of Progressive Scarring Trachoma in Ethiopia and Tanzania and Its Implications for Disease Control: Two Cohort Studies

**DOI:** 10.1371/journal.pntd.0003763

**Published:** 2015-05-13

**Authors:** Matthew J. Burton, Saul N. Rajak, Victor H. Hu, Athumani Ramadhani, Esmael Habtamu, Patrick Massae, Zerihun Tadesse, Kelly Callahan, Paul M. Emerson, Peng T. Khaw, David Jeffries, David C. W. Mabey, Robin L. Bailey, Helen A. Weiss, Martin J. Holland

**Affiliations:** 1 Faculty of Infectious and Tropical Diseases, London School of Hygiene & Tropical Medicine, London, United Kingdom; 2 National Institute for Health Research (NIHR) Biomedical Research Centre at Moorfields Eye Hospital and University College London (UCL) Institute of Ophthalmology, London, United Kingdom; 3 Department of Ophthalmology, Kilimanjaro Christian Medical Centre, Moshi, Tanzania; 4 The Carter Center, Addis Ababa, Ethiopia; 5 The Carter Center, Atlanta, Georgia, United States of America; 6 International Trachoma Initiative, Atlanta, Georgia, United States of America; 7 Medical Research Council (MRC) Laboratories, Fajara, The Gambia; 8 Medical Research Council (MRC) Tropical Epidemiology Group, London School of Hygiene & Tropical Medicine, London, United Kingdom; University of Cambridge, UNITED KINGDOM

## Abstract

**Background:**

Trachoma causes blindness through a conjunctival scarring process initiated by ocular *Chlamydia trachomatis* infection; however, the rates, drivers and pathophysiological determinants are poorly understood. We investigated progressive scarring and its relationship to conjunctival infection, inflammation and transcript levels of cytokines and fibrogenic factors.

**Methodology/Principal Findings:**

We recruited two cohorts, one each in Ethiopia and Tanzania, of individuals with established trachomatous conjunctival scarring. They were followed six-monthly for two years, with clinical examinations and conjunctival swab sample collection. Progressive scarring cases were identified by comparing baseline and two-year photographs, and compared to individuals without progression. Samples were tested for *C*. *trachomatis* by PCR and transcript levels of *S100A7*, *IL1B*, *IL13*, *IL17A*, *CXCL5*, *CTGF*, *SPARCL1*, *CEACAM5*, *MMP7*, *MMP9* and *CD83* were estimated by quantitative RT-PCR. Progressive scarring was found in 135/585 (23.1%) of Ethiopian participants and 173/577 (30.0%) of Tanzanian participants. There was a strong relationship between progressive scarring and increasing inflammatory episodes (Ethiopia: OR 5.93, 95%CI 3.31–10.6, p<0.0001. Tanzania: OR 5.76, 95%CI 2.60–12.7, p<0.0001). No episodes of *C*. *trachomatis* infection were detected in the Ethiopian cohort and only 5 episodes in the Tanzanian cohort. Clinical inflammation, but not scarring progression, was associated with increased expression of *S100A7*, *IL1B*, *IL17A*, *CXCL5*, *CTGF*, *CEACAM5*, *MMP7*, *CD83* and reduced *SPARCL1*.

**Conclusions/Significance:**

Scarring progressed in the absence of detectable *C*. *trachomatis*, which raises uncertainty about the primary drivers of late-stage trachoma. Chronic conjunctival inflammation appears to be central and is associated with enriched expression of pro-inflammatory factors and altered expression of extracellular matrix regulators. Host determinants of scarring progression appear more complex and subtle than the features of inflammation. Overall this indicates a potential role for anti-inflammatory interventions to interrupt progression and the need for trichiasis disease surveillance and surgery long after chlamydial infection has been controlled at community level.

## Author Summary

Blinding trachoma is believed to be the end result of a long-term progressive scarring process that is initiated by recurrent infection by the bacterium *Chlamydia trachomatis* starting in childhood. Scar tissue predominantly develops on the inner surface of the upper eyelids (conjunctiva). However, the rates, drivers and pathophysiological determinants are poorly understood. We investigated progressive scarring and its relationship to conjunctival infection, inflammation and transcript levels of cytokines and fibrogenic factors in two cohorts of adults in Tanzania and Ethiopia. These groups of people already had a degree of scarring and were regularly followed-up with over a two-year period. We found scarring progressed in about a quarter of people over this time. The progression was closely associated with episodes of conjunctival inflammation but not to the detection of *C*. *trachomatis* infection. This raises uncertainty about the primary drivers of late-stage trachoma. Chronic conjunctival inflammation appears to be central and is associated with enriched expression of pro-inflammatory factors and altered expression of extracellular matrix regulators. Host determinants of scarring progression appear more complex and subtle than the features of inflammation. Overall this indicates the likely need for trichiasis disease surveillance and surgery long after chlamydial infection has been controlled at community level.

## Introduction

Blinding trachoma is the end result of the scarring sequelae of recurrent ocular *Chlamydia trachomatis* infection. This obligate intracellular bacterium triggers prolonged inflammatory episodes in the conjunctival mucosa, which are believed to be central to the development of scarring [1]. However, a clear understanding of the immunopathological basis of this disease remains elusive. There is uncertainty over the relative importance of potential infectious drivers at different stages in the natural history of trachoma. Similarly, the protective and pathological host responses in relation to progressive scarring have not been fully characterised [1].

Typically, the disease starts in early childhood with a follicular-papillary conjunctivitis, which is associated with a mixed inflammatory cell infiltrate [2]. Scarring, primarily of the upper tarsal conjunctiva, generally begins to appear in adolescence, and accrues with age; it is characterised by thickened and disordered sub-mucosal collagen [3]. As this becomes more extensive the eyelid becomes distorted and turns in (entropion), which can lead to the eyelashes touching the eye (trichiasis). Blindness results from the corneal damage caused by trichiasis and secondary bacterial or fungal infection.

Trachoma is endemic in 51 countries and is estimated to be responsible for 2.2 million cases of blindness or low vision [4]. There is currently a major scale-up of the global control programme, which aims to eliminate the disease as a public health problem by the year 2020 [4]. Trachoma control focuses on the implementation of the SAFE Strategy: **S**urgery for trichiasis, **A**ntibiotics in the form of mass administration of azithromycin, **F**acial Cleanliness and **E**nvironmental Improvements to suppress the transmission of the infection [4].

In regions where the prevalence of *C*. *trachomatis* infection has been low for some years due to the implementation of the SAFE strategy or general socio-economic development, scarring complications such as incident trichiasis continue to present challenges for trachoma control [5]. This suggests that long term residents of endemic communities, who have been exposed to repeated chlamydial infection earlier in life, may continue to develop progressive conjunctival scarring, even when the prevalence of *C*. *trachomatis* has declined significantly. The basis for this progressive scarring disease is unknown and consequently biomarkers remain to be identified. Moreover, it has major implications for trachoma control programmes as it implies that incident trichiasis will continue to develop well beyond the 2020 elimination target set by the WHO, necessitating the ongoing provision of trichiasis surveillance and surgical services.

We investigated rates, drivers and pathophysiological determinants of progressive scarring trachoma in two parallel cohort studies of individuals with established trachomatous conjunctival scarring in Ethiopia and Tanzania, using a common methodology. Within each cohort, individuals who were found to have progressive scarring over a two-year period were compared to those with static disease. We investigated the rate of scarring progression associated with host determinants and clinical features.

## Materials and Methods

### Ethics statement

The Ethiopian study was reviewed and approved by the National Health Research Ethics Review Committee, Ministry of Science and Technology, Ethiopia, the London School of Hygiene and Tropical Medicine Ethics Committee (UK) and Emory University Institutional Review Board (Atlanta, USA). The Tanzanian study was reviewed and approved by the Tanzanian National Institute for Medical Research Ethics Committee, the Kilimanjaro Christian Medical Centre Ethics Committee and the London School of Hygiene and Tropical Medicine Ethics Committee. The studies adhered to the tenets of the Declaration of Helsinki. Potential participants were provided with both written and oral information in Amharic (Ethiopia) or Kiswahili (Tanzania). For those agreeing to participate, written informed consent was required prior to enrolment. If the participant was unable to read and write, the information sheet and consent form were read to them and their consent recorded by witnessed thumbprint, a form of consent approved by the Ethics Committees. These two-year cohort studies of progressive conjunctival scarring used the same methodology.

### Study design—Ethiopia

The Ethiopian study was a two-year longitudinal cohort with six-monthly follow-up, nested within a randomized trial of epilation versus eyelid surgery for the treatment of minor trichiasis in Amhara Region, Ethiopia. Recruitment was between March and June 2008. The results of this trial have been previously reported [6]. In brief, individuals aged 18 years or more with previously un-operated minor trachomatous trichiasis of the upper eyelid (<6 lashes touching the eye) were recruited. Trichiasis in the presence of upper tarsal conjunctival scarring was considered to be due to trachoma in the absence of another obvious cause for the trichiasis, such as trauma, malignancy, involutional changes or severe blepharitis. Following the baseline clinical assessment (described below), participants were randomized to one of two intervention groups: (i) posterior lamella tarsal rotation procedure or (ii) epilation [7]. Individuals in the epilation arm were each given two pairs of high quality, machine-manufactured epilation forceps with round edged tips and flat, opposing plates (Tweezerman). The patient and an accompanying adult with good near vision were trained to perform epilation. The population of this region of Ethiopia received annual mass azithromycin treatment before, during and after the two-year study period. For this sub-study of the pathogenesis of progressive trachomatous conjunctival scarring we only included people randomized to the epilation arm of the trial, who were identified retrospectively at the end of the two-year trial period, after the trial allocation had been unmasked. Individuals in the epilation arm with evidence of progressive scarring were identified and compared to individuals without progressive scarring.

### Study design—Tanzania

The Tanzanian study was also a two-year longitudinal cohort with six-monthly follow-up of individuals with trachomatous conjunctival scarring, but without trichiasis. It was conducted in three contiguous villages in Siha District, Kilimanjaro Region, Tanzania. We aimed to recruit all individuals aged 18 years or more with conjunctival scarring living within these communities. Recruitment was between March and June 2009. The methodology for identifying study participants has been previously described in reports of the baseline data [8–10]. Briefly, all households were visited, the adult population was enumerated and available adults were screened for the presence of trachomatous conjunctival scarring. Individuals with scarring were invited to join the cohort study. People with trichiasis or previous eyelid surgery were excluded. Those with current trichiasis were offered free surgery. Individuals with evidence of progressive scarring were identified and compared to individuals without progressive scarring.

### Clinical assessment and sample collection

Study participants were assessed at baseline, 6, 12, 18 and 24 months. They were examined for clinical signs of trachoma using 2.5x binocular loupes. On each occasion high resolution digital photographs were taken of the upper tarsal conjunctiva, for subsequent grading and comparison by a single ophthalmologist experienced in trachoma grading. Features were graded using the detailed WHO trachoma grading system with previously described modifications [6,8,11]. The tarsal conjunctival grading system is described in S1 Table [8]. Significant conjunctival inflammation was defined as the presence of papillary inflammation grades P2 or P3 of the detailed WHO Trachoma Grading System [11]. In both studies only one eye per person was included in the analysis for scarring progression and for the collection of conjunctival swab samples. In the Ethiopian study for individuals with bilateral trichiasis, the clinical trial study eye was randomly designated for inclusion as the outcome of epilation or surgery could be influenced by the laterality, although both eyes were treated. In the Tanzanian study the left eye was designated the study eye for all participants, for protocol simplicity.

On each occasion the ocular surface was anaesthetised with preservative-free proxymetacaine 0.5% eye drops (Minims, Chauvin Pharmaceuticals). Two conjunctival swab samples were collected from the upper tarsal conjunctival surface by making four horizontal passes across the conjunctival surface with a quarter turn between each pass (Dacron polyester-tipped swab: Hardwood Products Company). The first swab was collected for RNA isolation and placed directly into a tube containing 0.3ml of RNA*later* (Life Technologies). The second swab was collected for a DNA primed *C*. *trachomatis* PCR and was placed in a dry tube. Samples were kept on ice packs until frozen later the same day at—20°C. Long-term storage was at -80°C. The Ethiopian samples were transferred to Tanzania on dry ice for analysis.

### Progressors and Non-Progressors

After the completion of both studies, to determine whether there had been progression in conjunctival scarring, the baseline and 24-month tarsal conjunctival photographs were directly compared and graded by an ophthalmologist using a detailed scarring grading system, which stratifies severity on the proportion of the tarsal conjunctiva involved.[9] Individuals with progressive scarring (“Progressors”) were defined as those with photographic evidence of increased conjunctival scarring at 24-months. All other individuals did not have photographic evidence of progressive scarring (“Non-Progressors”). The ophthalmologist grading images was masked to the laboratory results.

Conjunctival gene expression was assessed in all “Progressors”. For comparison an equal number of “Non-Progressors” that were randomly selected from all of the non-progressing individuals within the same cohort. As scarring severity at baseline was considered to be an important determinant of the conjunctival gene expression profile, the Non-Progressors were frequency matched to the baseline scarring severity of the Progressors for both studies. In addition, in the Tanzanian cohort we found that the Progressors were significantly older than the Non-Progressors. Therefore, in the Tanzanian study, age was also included in the frequency matching random selection procedure.

For the Ethiopian cohort only Progressors and Non-Progressors who were examined at all five time points were included in the gene expression study. In Tanzania, the proportion that were examined at all five time points was slightly lower. Therefore, we also included people in the gene expression study who were seen on at least four out of the five time-points: 367 / 577 (63.6%) with both baseline and two-year assessments were eligible for inclusion in the analysis. As the primary focus of the study was the relationship between gene expression and the subsequent development of scarring by two-years, the final samples (24-month) were not analysed.

### Conjunctival gene expression analysis

All samples for both studies were tested at the Kilimanjaro Clinical Research Institute laboratory, Moshi, Tanzania, by the same assays, equipment and technical staff. Laboratory staff were masked to the clinical status. Total RNA was extracted from the swab samples using the RNeasy Micro Kit (Qiagen). Reverse transcription was performed using the QuantiTect Reverse Transcription Kit (Qiagen). The expression of eleven genes was estimated by multiplex real-time quantitative PCR, performed on a Rotor-Gene 6000 (Corbett Research, Cambridge, UK) using the QuantiTect Multiplex NoROX Kit (Qiagen). The choice of targets was informed by earlier studies of the conjunctival transcriptome of individuals with scarring trachoma in the same populations [10,12]. In these earlier studies we performed microarray experiments to determine the gene expression profile in the conjunctiva of scarring cases compared to controls. We selected genes for this study that showed significant up or down regulation in the microarray experiments and/or could plausibly be involved in pathways important in the inflammatory and scarring response. The targets studied were: interleukin-1β (*IL1B*), interleukin-13 (*IL13*), interleukin-17A (*IL17A*), psoriasin-1 (*S100A7*), chemokine (CXC) ligand-5 (*CXCL5*), matrix metalloproteinase-7 (*MMP7*), matrix metalloproteinase-9 (*MMP9*), connective tissue growth factor (*CTGF*), carcinoembryonic antigen-related cell adhesion molecule 5 (*CEACAM5*), CD83 and SPARC-like 1 (*SPARCL1*). Multiplex assays of up to four separate targets (including *HPRT-1* as the reference gene) were designed by Sigma Life Science (www.Sigma.com/designmyprobe) using Beacon Designer 7.60 (Premier Biosoft International, Palo Alto, CA, USA). The thermal cycle conditions were: 95°C for 15 minutes, followed by 45 cycles of (1) denaturation at 94°C for 30 seconds, (2) annealing and extension at 60°C for 30 seconds. Fluorescence data was acquired at the end of each cycle. The relative efficiency of the component reactions was assessed using standards containing all targets in a ten-fold dilution series. Reactions were performed in duplicate, in a total volume of 25μl, which contained 2μl of sample or standard. Probe and primer sequences are available on request. The paired dry swab samples were tested for *C*. *trachomatis* DNA using a PCR-based assay (Amplicor CT/NG Test; Roche) with previously described modifications [13].

### Data analysis

We estimated that a sample size of 100 Progressors and 100 Non-Progressors would have 80% power and 95% confidence to detect a factor with an odds ratio of 2.5 that is present in 50% of the Non-Progressors.

Data were managed in Access (Microsoft) and analysed in STATA 12 (StataCorp). The analysis was unmatched because the non-progressors were frequency matched rather than individually matched. BMI was used as a marker for nutritional status. The transcript abundances were standardised relative to that of *HPRT-1* in the same reaction using the ΔΔC_T_ method and were log_10_ transformed. Together these correct for variations in the total mRNA recovered from the swabs and fit the data to a normal distribution [14]. As Progressors and Non-Progressors were of comparable age, sex and baseline scarring severity, the relative level of expression of each target was compared between them using unadjusted unpaired *t*-tests.

Multivariable linear regression models were fitted for the expression level of each target at all time points when measured and the following potential explanatory variables: sex (female), age (in years), conjunctival inflammation (P2/P3) at the same time point and scarring progression. We combined the data from the four time points (Baseline, 6, 12 and 18 months), using random-effects linear regression models. A stepwise selection process was performed to fit each model, retaining terms if the *p*-value in the model was <0.2. To adjust for multiple comparisons we used the Benjamini and Hochberg approach, assuming a false discovery rate (FDR) of 5% [15].

To try to identify possible gene expression correlates or biomarkers of progressive scarring we interrogated the data using a number of alternative bioinformatics techniques. Both Ethiopian and Tanzanian datasets were examined at each time point using unsupervised hierarchical clustering of expression heat maps and topologically embedded graphs, supervised classification using support vector machine with 10-fold cross validation and multivariate differential correlation of co-expression networks [16,17].

## Results

### Ethiopian participants

**Fig 1:**
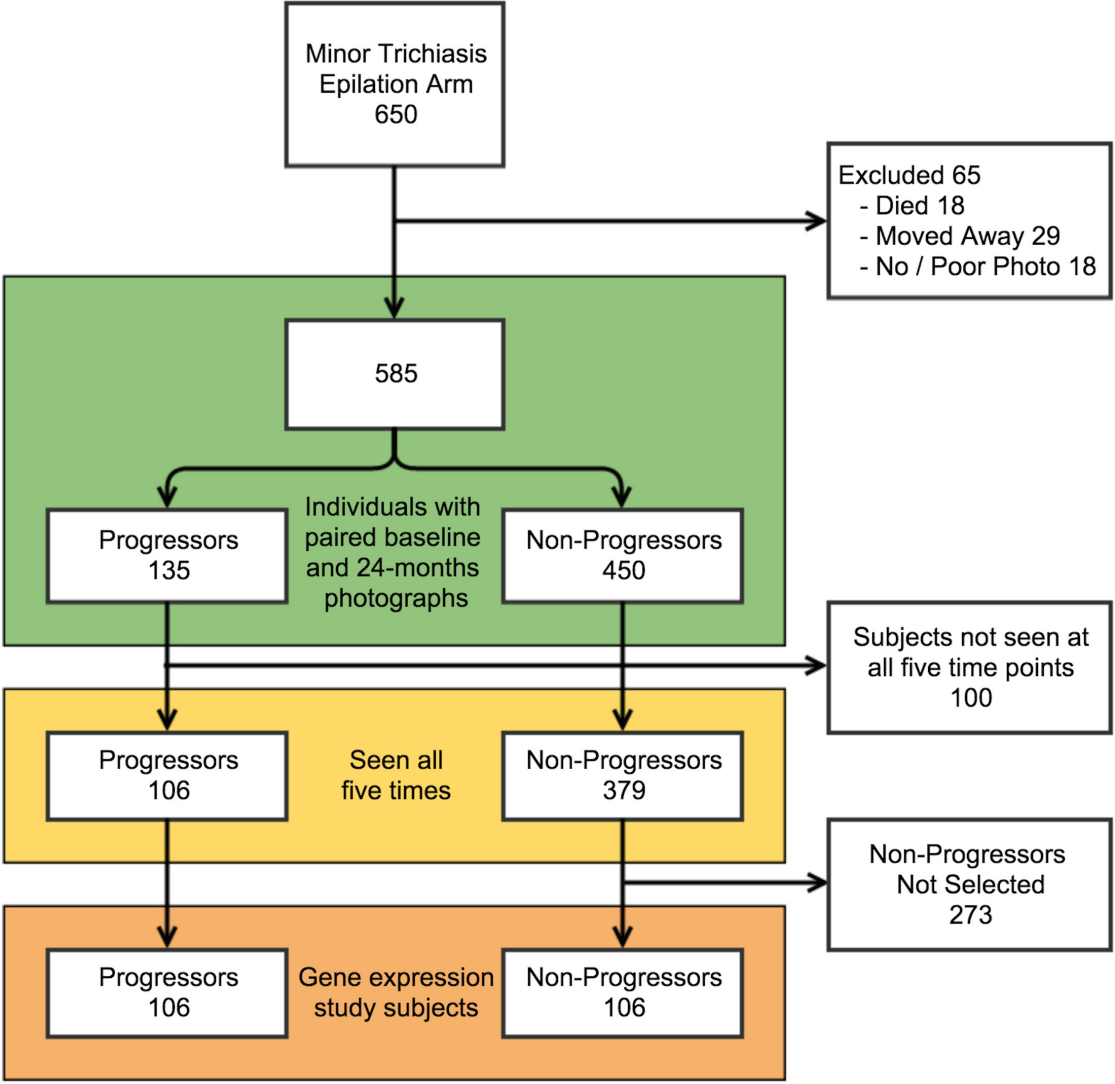
Ethiopian cohort participant flow.

A total of 1300 people with minor trichiasis were recruited into the Ethiopian trial, of whom 650 were randomly allocated to the epilation arm [6]. Of these 650 people, 585 had paired tarsal conjunctival photographs from both baseline and 24-months and were included in the analysis of clinical progression (Fig 1). The mean duration of follow-up was 677 days (S.D. 39 days). There were 65 individuals who were not seen at 24-months (died or moved away) or whose paired photographs were of insufficient quality for comparison. These 65 who were not included were slightly older (mean age: 53.4 years vs 50.4 years), however this difference was not significant (t-test, p = 0.11), and had a similar proportion that were female as those included (64.6% vs 63.8%; p = 0.89).

### Tanzanian participants

The three Tanzanian villages had a total adult population of 3626 people at the time of the census, of whom 2418 (67%) were examined. Of those not seen, 711 (19.6%) were absent at the time of the census (despite two visits), 347 (9.6%) were temporarily resident elsewhere, and 150 (4.1%) refused examination. Of the 2418 who were examined: 862 (35.6%) had trachomatous conjunctival scarring, 1520 (62.9%) did not have scarring and 36 (1.5%) were excluded due to the presence of trichiasis or previous eyelid surgery.

**Fig 2:**
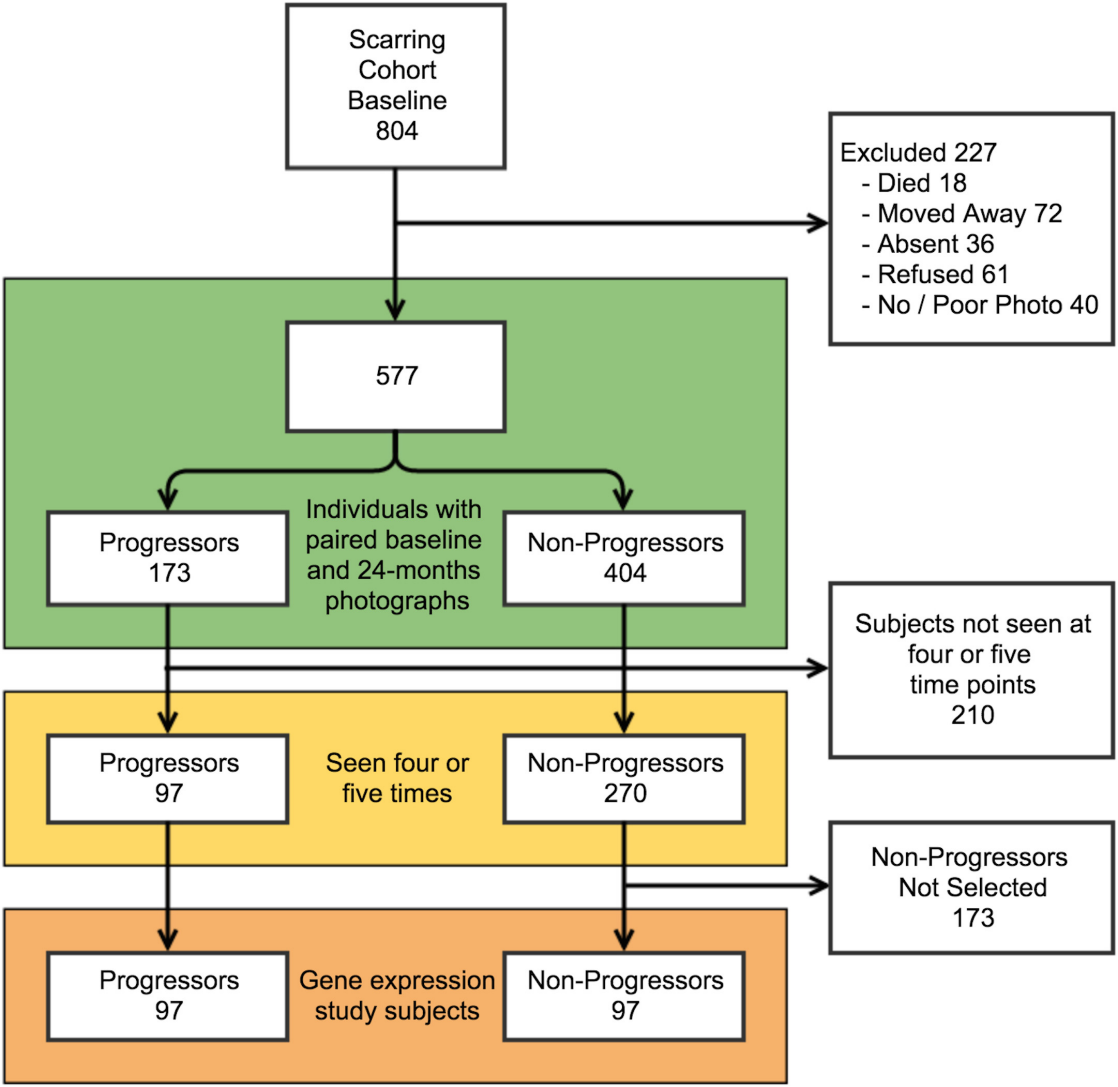
Tanzanian cohort participant flow.

A total of 804 Tanzanian individuals with conjunctival scarring agreed to participate in the longitudinal study. Of these 804 people, 577 had paired tarsal conjunctival photographs from both baseline and 24-months and were included in the analysis of clinical progression (Fig 2). The mean duration of follow-up was 688 days (S.D. 40 days). There were 227 individuals who were not seen at 24-months (died, moved away, absent or refused) or whose paired photographs were of insufficient quality for comparison. These 227 who were not included were slightly younger (mean age: 43.3 years vs 45.9 years; t-test, p = 0.062) and had a similar proportion who were female compared to those included (59.5% vs 62.7%; p = 0.39).

### Ethiopian cohort—Progression in conjunctival scarring

**Fig 3:**
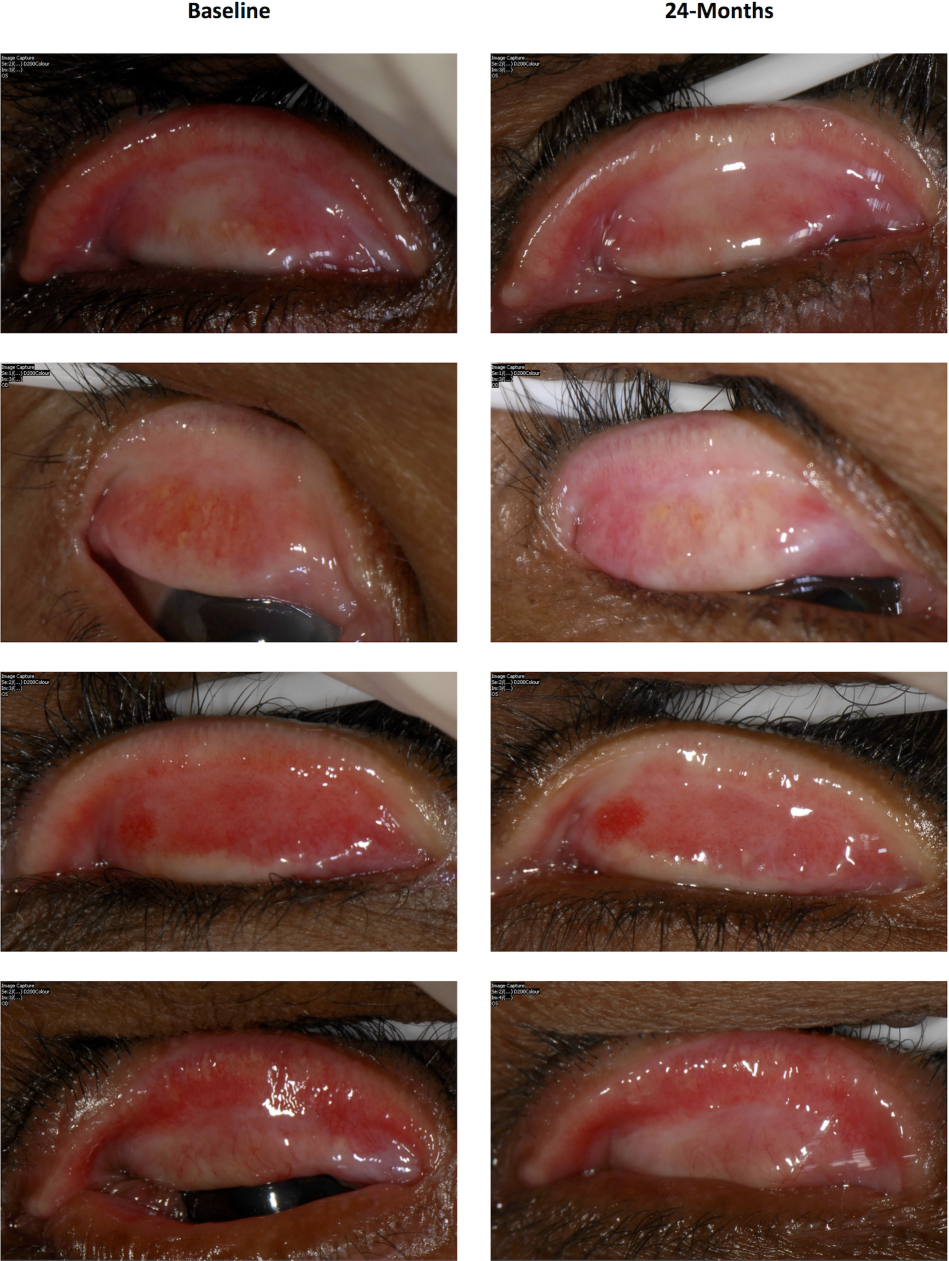
Examples of paired photographs from individuals showing signs of increasing upper tarsal conjunctival scarring between baseline and 24-months.

Between baseline and 24-months there was visible progression of the upper tarsal conjunctival scarring in 135 / 585 (23.1%) of the Ethiopian participants (Fig 1 and Fig 3). There was no association between scarring progression and age, gender or BMI (Table 1). Progressors had slightly less marked baseline scarring than Non-Progressors (Table 1). There was an association between the presence of clinical inflammation (P2/P3) on each occasion and progression of scarring by 24-months (Table 1). Clinical inflammation was present on at least one occasion in 71.9% of Progressors and 42.7% of Non-Progressors (p<0.0001). There was a strong relationship between progressive scarring and an increasing proportion of occasions (from none to all) when clinical inflammation was observed (OR 5.93, 95%CI 3.31–10.6, p<0.0001).

**Table 1:** Baseline and intervening time point demographic and clinical characteristics of (A) 585 Ethiopian participants and (B) 577 Tanzanian participants included in the analysis of progression of conjunctival scarring.

Characteristic	Progressors		Non-Progressors		P-value
**A) ETHIOPIA**	**N = 135**		**N = 450**		
Gender (female)	93	(68.9%)	280	(62.2%)	0.16
Age at baseline, mean (SD)	49.4	(13.4)	50.6	(14.5)	0.42
BMI at baseline, mean (SD)	20.1	(2.4)	20.0	(2.3)	0.73
Scarring severity at baseline					<0.0001
1a	0	-	1	(0.2%)	
1b	6	(4.4%)	4	(0.9%)	
1c	20	(14.8%)	21	(4.7%)	
2	107	(79.3%)	342	(76.0%)	
3	2	(1.5%)	82	(18.2%)	
Clinically inflamed (P2/P3)					
Baseline	71 / 135	(52.6%)	136 / 450	(30.2%)	<0.0001
6-months	26 / 95	(27.4%)	50 / 341	(14.7%)	0.0039
12-months	58 / 130	(44.6%)	84 / 430	(19.4%)	<0.0001
18-months	29 / 131	(22.1%)	60 / 436	(13.8%)	0.021
Clinically inflamed, 1+ episode	97	(71.9%)	192	(42.7%)	<0.0001
Proportion of examinations with inflammation (%)					<0.0001
0%	38	(28.1%)	258	(57.3%)	
25%	28	(20.7%)	77	(17.1%)	
33%	11	(8.1%)	25	(5.6%)	
50%	19	(14.1%)	32	(7.1%)	
66%	9	(6.7%)	16	(3.6%)	
75%	11	(8.1%)	18	(4.0%)	
100%	19	(14.1%)	24	(5.3%)	
**B) TANZANIA**	**N = 173**		**N = 404**		
Gender (female)	115	(66.5%)	247	(61.1%)	0.23
Age at baseline, mean (SD)	50.9	(17.7)	43.8	(16.9)	<0.0001
BMI at baseline, mean (SD)	21.6	(3.1)	21.8	(2.9)	0.47
Scarring severity at baseline					<0.0001
1a	45	(26.0%)	170	(42.1%)	
1b	63	(36.4%)	170	(42.1%)	
1c	45	(26.0%)	47	(11.6%)	
2	14	(8.1%)	12	(3.0%)	
3	6	(3.5%)	5	(1.2%)	
Clinically inflamed (P2/P3)					
Baseline	46 / 173	(26.6%)	55 / 404	(13.6%)	0.0002
6-months	21 / 152	(13.8%)	15 / 357	(14.7%)	0.0001
12-months	13 / 143	(9.1%)	16 / 335	(4.8%)	0.070
18-months	6 / 135	(4.4%)	3 / 325	(1.0%)	0.013
Clinically inflamed, 1+ episode	53 / 173	(30.6%)	69 / 404	(17.1%)	0.0003
Proportion of examinations with inflammation (%)					0.0011
0%	120	(69.4%)	335	(82.9%)	
25%	19	(11.0%)	37	(9.2%)	
33%	8	(4.6%)	13	(3.2%)	
50%	8	(4.6%)	9	(2.2%)	
66%	3	(1.7%)	2	(0.5%)	
75%	5	(2.9%)	2	(0.5%)	
100%	10	(5.8%)	6	(1.5%)	

### Tanzanian cohort—Progression in conjunctival scarring

Overall, the Tanzanian cohort had less severe scarring compared to the Ethiopian cohort (Table 1; p<0.0001). There was visible progression of the upper tarsal conjunctival scarring in 173 / 577 (30.0%) Tanzanian participants between baseline and 24-months (Fig 2). There was no association between scarring progression and gender or BMI (Table 1). The Progressors were significantly older than the Non-Progressors (p<0.0001). Progressors had slightly more marked baseline scarring than Non-Progressors (Table 1). There was an association between the presence of clinical inflammation (P2/P3) and progression of scarring by 24-months (Table 1). Clinical inflammation was present on at least one occasion in 30.6% of Progressors and 17.1% of Non-Progressors (p = 0.0003). There was a strong relationship between progressive scarring and an increasing proportion of occasions (from none to all) when clinical inflammation was observed (OR 5.76, 95%CI 2.60–12.7, p<0.0001).

### Selection of Ethiopian participants for gene expression analysis

Of the 585 participants assessable for scarring progression, there were 100 individuals who were not seen on all occasions (Fig 1). The 485 people seen at all five time points were eligible for inclusion in the gene expression study. Amongst these there were 106 (21.8%) Progressors and 379 (78.2%) Non-Progressors (Fig 1). The Non-Progressors included a greater proportion of people with more severe scarring at baseline. As the expression of targets may be correlated with disease severity, we frequency matched a random selection of 106 Non-Progressors to the baseline scarring severity score of the 106 Progressors (Fig 1). The 106 Progressors and 106 selected Non-Progressors in the gene expression analysis had comparable ages, proportion of females and BMI (Table 2). The Progressors had more baseline conjunctival inflammation (Table 2).

**Table 2:** Baseline and intervening time point demographic and clinical characteristics of individuals included in the gene expression analysis.

Characteristic	Progressors		Non-Progressors		P-value
**A) ETHIOPIA**	**N = 106**		**N = 106**		**P-value**
Gender (female)	72	(67.9%)	64	(60.4%)	0.25
Age at baseline, mean (SD)	50.2	(13.0)	50.8	(15.1)	0.74
BMI at baseline, mean (SD)	20.0	(2.4)	20.1	(2.3)	0.73
Lash count, mean (SD)	1.5	(1.2)	1.5	(1.1)	0.91
Scarring severity at baseline^A^					
1a	0	-	0	-	
1b	1	(1.0%)	1	(1.0%)	
1c	14	(13.2%)	14	(13.2%)	
2	89	(84.0%)	89	(84.0%)	
3	2	(1.9%)	2	(1.9%)	
Clinically inflamed (P2/P3)					
Baseline	52 / 106	(49.1%)	34 / 106	(32.1%)	0.012
6-months	23 / 87	(26.4%)	17 / 94	(18.1%)	0.18
12-months	45 / 106	(42.6%)	24 / 106	(22.6%)	0.0021
18-months	19 / 104	(18.3%)	13 / 104	(12.5%)	0.25
Clinically inflamed, 1+ episode	74	(69.8%)	46	(43.4%)	0.0001
Proportion of examinations with inflammation (%)					0.0028
0%	32	(30.2%)	60	(56.6%)	
25%	27	(25.5%)	19	(17.9%)	
33%	6	(5.7%)	0	-	
50%	19	(15.1%)	10	(9.4%)	
66%	4	(3.8%)	5	(4.7%)	
75%	11	(10.4%)	7	(6.6%)	
100%	10	(9.4%)	5	(4.7%)	
**B) TANZANIA**	**N = 97**		**N = 97**		**P-value**
Gender (female)	64	(66.0%)	55	(56.7%)	0.19
Age at baseline, mean (SD)	51.8	(17.9)	52.8	(18.5)	0.69
BMI at baseline, mean (SD)	21.3	(2.9)	21.7	(3.3)	0.36
Scarring severity at baseline ^B^					
1a	10	(10.3%)	10	(10.3%)	
1b	32	(33.0%)	33	(34.0%)	
1c	34	(35.1%)	38	(39.2%)	
2	12	(12.4%)	9	(9.3%)	
3	9	(9.2%)	7	(7.2%)	
Clinically inflamed (P2/P3)					
Baseline	30 / 97	(30.9%)	17 / 97	(17.5%)	0.029
6-months	16 / 94	(17.0%)	7 / 94	(7.5%)	0.045
12-months	12 / 89	(13.5%)	1 / 81	(1.2%)	0.0026
18-months	5 / 83	(6.0%)	0 / 88	-	0.019
Clinically inflamed, 1+ episode	33 / 97	(34.0%)	22 / 97	(22.7%)	0.08
Proportion of examinations with inflammation (%)					0.051
0%	64	(66.0%)	75	(77.3%)	
25%	10	(10.3%)	11	(11.3%)	
33%	7	(7.2%)	8	(8.3%)	
50%	3	(3.1%)	2	(2.1%)	
66%	2	(2.1%)	1	(1.0%)	
75%	5	(5.2%)	0	-	
100%	6	(6.2%)	0	-	

(A) 212 Ethiopian participants; non-progressors were frequency matched on baseline scarring severity. (B) 194 Tanzanian participants; non-progressors were frequency matched on baseline scarring severity and age.

^A^ Non-Progressors were frequency matched on baseline scarring to the Progressors.

^B^ Non-Progressors were frequency matched on baseline scarring and age to the Progressors.

### Selection of Tanzanian participants for gene expression analysis

Of the 577 participants assessable for scarring progression, 210 individuals were not seen on all occasions (Fig 2). The 367 people seen at four or five time points were eligible for inclusion in the gene expression study. Amongst these there were 97 (26.4%) Progressors and 270 (73.6%) Non-Progressors (Fig 2). Unlike the Ethiopian Progressors, the Tanzanian Progressors had a greater proportion of people with more severe scarring at baseline and tended to be older than the Non-Progressors (Table 1). As the expression of targets may be correlated with disease severity and age, we frequency matched a random selection of 97 Non-Progressors to the baseline scarring severity score and age (in five-year bands) of the 97 Progressors (Fig 2). The 97 Progressors and 97 selected Non-Progressors in the gene expression analysis had comparable ages, proportion of females and BMI (Table 2). The Progressors had more baseline conjunctival inflammation (Table 2).

### Gene expression analysis

In the Ethiopian cohort a total of 848 samples were collected from the 212 participants in the gene expression study at four time points (baseline, 6, 12 and 18 months). There were 13 / 848 samples in which we were unable to detect the expression of any of the eleven targets, probably due to an inadequate sample or failed extraction. In the Tanzanian cohort a total of 719 samples were collected from the 194 participants in the gene expression study. There were 70 / 97 Progressors and 69 / 97 Non-Progressors in the Tanzanian cohort who were sampled on all four occasions (baseline, 6, 12 and 18 months). A further 27 Progressors and 28 Non-Progressors were sampled on three occasions. Two samples from the Progression group were lost and there were 5 / 719 samples in which we were unable to detect the expression of any of the eleven targets, probably due to an inadequate sample or failed extraction.

The expression level for each target was compared by unpaired t-test between the Progressors and the Non-Progressors for each of the time points in both cohorts. No significant (FDR adjusted) associations were found between scarring progression and the expression of any target in either cohort (Table 3). In the Ethiopian cohort clinical inflammation (P2/P3) was consistently associated with increased expression of *S100A7*, *IL1B*, *IL17A*, *CXCL5*, *CTGF*, *MMP7*, *CD83* and reduced expression of *SPARCL1* (Table 4). In the Tanzanian cohort clinical inflammation was consistently associated with increased expression of *S100A7*, *IL17A*, *CXCL5*, *MMP7* and *CEACAM5* (Table 4). The mRNA transcript in both cohorts with the greatest fold change was *CXCL5*.

**Table 3:** Gene expression levels of specific targets in Scarring Progressors relative to Non-Progressors in Ethiopia and Tanzania.

Target	Baseline		6-months		12-months		18-months	
	FC	P-value	FC	P-value	FC	P-value	FC	P-value
**ETHIOPIA**								
*S100A7*	1.20	0.44	1.31	0.23	1.27	0.22	1.09	0.65
*IL1B*	1.44	0.17	1.01	0.98	1.08	0.75	1.10	0.71
*IL13*	1.06	0.78	1.28	0.18	1.56	0.002	1.39	0.07
*IL17A*	1.31	0.20	1.10	0.70	1.21	0.34	1.00	0.99
*CXCL5*	1.21	0.59	1.27	0.48	1.29	0.46	1.08	0.82
*CTGF*	1.14	0.66	0.70	0.21	1.20	0.52	1.08	0.80
*CEACAM5*	1.08	0.61	1.26	0.14	1.24	0.17	1.08	0.60
*MMP7*	1.25	0.15	1.19	0.24	1.18	0.26	1.12	0.44
*MMP9*	1.43	0.01	0.86	0.23	1.06	0.59	1.05	0.66
*CD83*	1.11	0.31	1.06	0.57	1.00	0.96	1.12	0.25
*SPARCL1*	1.06	0.78	1.21	0.23	1.22	0.17	1.01	0.97
**TANZANIA**								
*S100A7*	2.00	0.0027	1.10	0.63	1.47	0.08	1.71	0.022
*IL1B*	2.05	0.017	1.00	0.98	1.99	0.009	1.42	0.11
*IL13*	1.45	0.18	1.23	0.50	0.78	0.35	1.30	0.24
*IL17A*	1.55	0.046	0.96	0.83	1.43	0.06	1.12	0.86
*CXCL5*	1.46	0.10	1.18	0.46	1.20	0.44	1.55	0.08
*CTGF*	1.21	0.61	1.05	0.89	1.92	0.09	1.21	0.66
*CEACAM5*	1.37	0.08	1.03	0.87	1.22	0.22	1.20	0.23
*MMP7*	1.18	0.22	1.17	0.20	1.33	0.053	1.07	0.63
*MMP9*	1.02	0.88	1.29	0.07	1.10	0.52	1.15	0.37
*CD83*	1.35	0.0015	1.04	0.69	1.25	0.07	1.03	0.73
*SPARCL1*	0.93	0.78	0.93	0.63	0.89	0.60	1.06	0.69

FC fold change; P values for unpaired *t* test. Using the Benjamini and Hochberg approach only tests with a p-value below 0.0011 in the Ethiopian study and a p-value below 0.0011 in the Tanzanian study have a False Discovery Rate of <5%.

**Table 4:** Gene expression levels of specific targets in Ethiopians and Tanzanians with clinical inflammation (P2/P3) relative to non-inflamed (P0/P1) individuals.

Target	Baseline		6-months		12-months		18-months	
	FC	P-value	FC	P-value	FC	P-value	FC	P-value
**ETHIOPIA**								
*S100A7*	2.44	0.0001	4.69	<0.0001	2.53	<0.0001	1.15	0.58
*IL1B*	1.71	0.049	7.19	<0.0001	2.49	0.0008	1.43	0.30
*IL13*	0.84	0.39	1.57	0.072	1.24	0.17	1.43	0.17
*IL17A*	2.04	0.0008	3.20	0.0001	2.05	0.0007	1.22	0.47
*CXCL5*	8.92	<0.0001	12.91	<0.0001	4.18	0.0001	2.17	0.11
*CTGF*	3.10	0.0001	6.14	<0.0001	2.93	0.0002	2.15	0.08
*CEACAM5*	1.76	0.0001	2.93	<0.0001	1.81	0.0002	1.32	0.14
*MMP7*	1.33	0.07	1.77	0.0045	1.59	0.0027	1.33	0.16
*MMP9*	0.80	0.12	1.20	0.27	1.13	0.30	1.02	0.89
*CD83*	1.22	0.046	1.79	<0.0001	1.48	0.0002	1.29	0.06
*SPARCL1*	0.50	0.0001	0.50	0.0007	0.70	0.0172	0.56	0.030
**TANZANIA**								
*S100A7*	1.92	0.017	1.81	0.05	1.69	0.20	6.44	0.0071
*IL1B*	2.28	0.022	1.60	0.26	2.40	0.08	1.36	0.64
*IL13*	1.02	0.95	1.03	0.95	1.26	0.64	2.35	0.29
*IL17A*	2.93	<0.0001	1.70	0.05	4.17	0.0001	2.04	0.69
*CXCL5*	3.16	<0.0001	2.60	0.0053	4.01	0.0013	6.98	0.0080
*CTGF*	1.21	0.67	2.65	0.08	2.86	0.15	14.13	0.039
*CEACAM5*	2.60	<0.0001	2.64	0.0001	1.83	0.046	3.82	0.0024
*MMP7*	1.55	0.0057	1.77	0.0026	1.88	0.022	2.06	0.08
*MMP9*	1.07	0.70	1.30	0.22	1.05	0.86	2.20	0.09
*CD83*	1.25	0.047	1.44	0.0085	1.98	0.0025	1.68	0.06
*SPARCL1*	1.46	0.19	0.41	0.0005	0.68	0.45	0.48	0.16

FC fold change; P values for unpaired *t* test. Using the Benjamini and Hochberg approach only tests with a p-value below 0.030 in the Ethiopian study and a p-value below 0.030 in the Tanzanian study have a False Discovery Rate of <5%.

Multivariable linear regression models (random-effects model) for each target for all the time-points simultaneously were fitted for a combination of the potential determinants (Table 5). In these, progressive scarring was not found to be associated with any of the targets in the Ethiopian cohort but was associated with increased *IL1B* expression and borderline increased *S100A7* expression in the Tanzanian cohort. Conjunctival inflammation was associated with increased *S100A7*, *IL1B*, *IL17A*, *CXCL5*, *CTGF*, *CEACAM5*, *CD83* and reduced expression of *SPARCL1* in the Ethiopian cohort and increased expression of *S100A7*, *CXCL5* and CEACAM5 in the Tanzanian cohort.

**Table 5:** Multivariable linear regression models (random effects) for the expression of each target for all four time points combined.

		Ethiopia		Tanzania	
Target	Variable	Fold Change	P-value ^a^	Fold Change	P-value ^b^
***S100A7***	Gender (female)	1.38	0.049	-	-
	Age (years)	1.02	<0.0001	1.02	<0.0001
	Inflammation (P2/P3)	2.28	<0.0001	1.69	0.0009
	Progressive Scarring	-	-	1.43	0.027
***IL1B***	Gender (female)	1.25	0.18	-	-
	Age (years)	-	-	0.99	0.028
	Inflammation (P2/P3)	2.61	<0.0001	-	-
	Progressive Scarring	-	-	1.54	0.0067
***Il13***	Gender (female)	-	-	-	-
	Age (years)	-	-	0.98	0.0003
	Inflammation (P2/P3)	-	-	-	-
	Progressive Scarring	1.27	0.034	-	-
***IL17A***	Gender (female)	1.75	0.0004	-	-
	Age (years)	-	-	0.99	0.014
	Inflammation (P2/P3)	1.87	<0.0001	-	-
	Progressive Scarring	-	-	-	-
***CXCL5***	Gender (female)	2.90	0.0001	1.59	0.0061
	Age (years)	1.02	0.0023	-	-
	Inflammation (P2/P3)	3.20	<0.0001	2.22	<0.0001
	Progressive Scarring	-	-	-	-
***CTGF***	Gender (female)	1.32	0.19	-	-
	Age (years)	1.02	0.0007	-	-
	Inflammation (P2/P3)	3.13	<0.0001	1.72	0.08
	Progressive Scarring	-	-	-	-
***CEACAM5***	Gender (female)	1.52	0.0002	-	-
	Age (years)	1.01	0.010	-	-
	Inflammation (P2/P3)	1.36	0.0001	1.55	0.0003
	Progressive Scarring	-	-	-	-
***MMP7***	Gender (female)	-	-	-	-
	Age (years)	1.01	0.0025	0.99	0.0006
	Inflammation (P2/P3)	1.32	0.085	1.19	0.048
	Progressive Scarring	-	-	-	-
***MMP9***	Gender (female)	-	-	-	-
	Age (years)	-	-	0.99	0.023
	Inflammation (P2/P3)	-	-	-	-
	Progressive Scarring	0.86	0.050	-	-
***CD83***	Gender (female)	1.21	0.010	-	-
	Age (years)	1.01	0.040	0.99	0.0008
	Inflammation (P2/P3)	1.32	<0.0001	-	-
	Progressive Scarring	-	-	1.15	0.048
***SPARCL1***	Gender (female)	0.65	0.0002	1.01	0.022
	Age (years)	0.99	0.016	-	-
	Inflammation (P2/P3)	0.54	<0.0001	0.76	0.085
	Progressive Scarring	-	-	-	-

^a^ For the Ethiopian analysis, using the Benjamini and Hochberg approach, only tests with a p-value below 0.034 have a False Discovery Rate of <5%.

^b^ For the Tanzanian analysis, using the Benjamini and Hochberg approach, only tests with a p-value below 0.014 have a False Discovery Rate of <5%.

### Tests for biomarkers of progressive scarring

**Fig 4:**
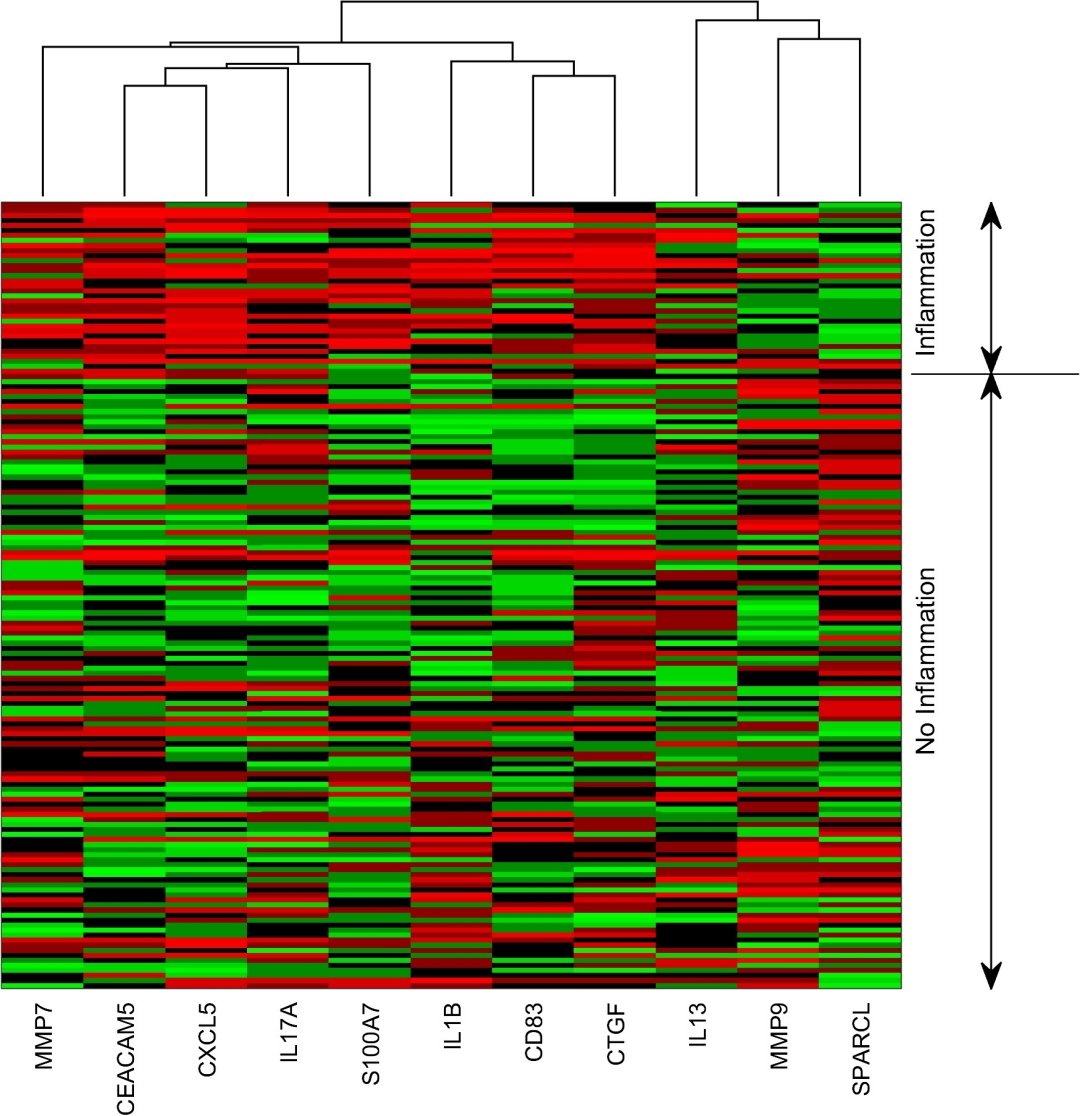
Gene expression heat map for the Ethiopian cohort at six-months, subdivided into those with and without clinical inflammation. Expression data was log_10_ transformed and then standardised to a mean of 0 and a standard deviation of 1. Colour coding: red represents values above the mean, black represents the mean and green represents values below the mean. Values above a standardized value of 3 are all coloured red and those below -3 are all coloured green. Data are clustered according to gene via hierarchical clustering using an average linkage. Rows are not clustered and simply divided into two categories, inflamed and non-inflamed.

Heat maps and embedded graphs visually confirmed the results of the formal methods used in the hypothesis-based tests: for inflammation (with the exception of *IL-13* and *MMP9*), the measured targets are good discriminators of inflammation (Fig 4). For scarring, none of the gene expression factors or combination of factors that were measured discriminated those individuals with a progressive scarring phenotype. Supervised classification using support vector machine was unable to find evidence that gene expression could predict or classify progressive scarring (error rates between 16% and 30% dependent on the time point). Differential correlation of gene-sets was also unable to identify any significant eigengene (module/pathway) or biomarker (critical node) indicative of progressive scarring.

### *C*. *trachomatis* detection

All 212 participants in the Ethiopian cohort had samples tested for *C*. *trachomatis* by PCR from baseline, 6, 12 and 18-months. All 848 samples tested negative. In the Tanzanian cohort we tested the baseline samples from all 804 participants; 4 of these were positive. At 6, 12 and 18-months only the samples from the 194 participants in the gene expression study were tested; one sample from the 12-month assessment tested positive. All other samples were negative.

## Discussion

This study found about a quarter of individuals with established conjunctival scarring in two distinct populations had disease progression over a two-year period. This progression was not associated with detectable *C*. *trachomatis* infection in either cohort. This observation has significant implications for trachoma control programmes, as it suggests that in populations where *C*. *trachomatis* infection was previously thought to be endemic but can now only be detected infrequently, scarring sequelae still progress. Therefore, it is probable that new cases of trichiasis will continue to develop in the years beyond 2020, as the population ages. This will necessitate the on going provision of surveillance for trichiasis and surgical services for an unknown period of time. Given that comparable results were obtained for two separate populations, it would seem likely that the conclusions hold for other trachoma endemic populations.

The study also provides prospective evidence that progression of trachomatous conjunctival scarring is associated with clinically apparent mucosal inflammation. Moreover, this association with progressive scarring appears to be stronger in those with more frequent inflammatory episodes. There are few prospective studies on progressive trachomatous conjunctival scarring with detailed follow-up. This reflects the challenge of studying this slowly progressive disease process, which can take many years to develop. However, the consistent observation is that generalised hyperaemia and papillary inflammation (rather than follicular inflammation) is associated with scarring progression.

Two earlier prospective studies have examined the progression of pre-existing conjunctival scarring. In the first, from Tunisia, a group of 213 people, with and without pre-existing scarring, living in a trachoma endemic village were examined on two occasions 14 years apart [18]. The development of severe scarring (C3) was strongly associated with the presence at baseline of severe inflammatory trachoma (Relative Risk 18) and pre-existing mild or moderate baseline scarring (Relative Risk 19). Progression to C3 at 14 years occurred in 19% of those with mild baseline scarring (C1) and 69% of those with moderate baseline scarring (C2). In the second study, from Tanzania, 85 individuals with scarring at baseline were reassessed at five years [19]. Photograph grading was used (without direct comparison of paired baseline and five-year images) and 47% were judged to have progressed. The relationship with inflammation was not reported.

Other studies have examined the development of incident scarring in people without scarring at baseline. An earlier study in Tanzanian women found the five-year incidence of scarring was age dependent (relationship to inflammation not reported): 3.2% in 15–19 year olds and 14.3% in 55–59 year olds [20]. In a cohort of Tanzanian children followed for seven years, “constant severe trachoma” during the first year was associated with subsequent scarring [21]. Finally, in a second cohort study of Tanzanian children, again “constant severe trachoma” was associated with development of incident scarring [22].

It is generally believed that the major driver for progressive scarring is repeated exposure to *C*. *trachomatis*, which provokes an inflammatory response, characterised by the signs observed in the conjunctiva. Numerous studies have demonstrated a clear association between the signs of active inflammatory trachoma (TF/TI) in children and the detection of *C*. *trachomatis*. However, in contrast, the direct evidence linking progression of established scarring to *C*. *trachomatis* is limited. The only human study that has assessed the development of scarring in relation to chlamydial infection was a five-year cohort of 189 Tanzanian children who mostly had no baseline scarring [22]. In this study children who had chlamydial infection detected by PCR on several occasions during the first 18 months were at increased risk of developing incident scarring by five years compared to those with no or only sporadic infection.

In contrast to the study of Tanzanian children, the present longitudinal studies of Ethiopian and Tanzanian adults with established scarring did not detect any (Ethiopia), or only very few (Tanzania), episodes of *C*. *trachomatis* infection in multiple samples collected over a two-year period. This was despite half of the tested participants having progressive scarring. In Ethiopia, the region where the study was based has historically experienced hyperendemic active trachoma in children and the trichiasis prevalence is one of the highest known [23]. However, for several years prior to this study the regional trachoma control programme had conducted synchronous mass drug administration (MDA) with azithromycin across a wide geographical area with only moderate reductions in active trachoma reported. It is known that following the introduction of MDA the relationship between active follicular trachoma (TF) and the presence of detectable infection is much more variable [5,24–26]. Therefore, the underlying rate of infection in the population may have declined, explaining why no cases of infection were detected in the Ethiopian cohort. In Tanzania, the villages where the cohort was recruited had never received MDA for trachoma control, however, the impression is that the current prevalence of active trachoma and chlamydial infection in this area is relatively low, based on data from neighbouring communities [27].

It is hoped that an underlying reduction in the community prevalence of *C*. *trachomatis* due to azithromycin MDA would reduce the rates of incident and progressive scarring. Although in these populations despite an apparently very low frequency of chlamydial infection in adults with scarring, the disease still progressed. In addition, it is possible that azithromycin may also have a direct anti-inflammatory effect, possibly through enhancing neutrophil apoptosis, which could in turn slow scarring [28].

The absence of an association between progressive scarring and current *C*. *trachomatis* infection could have a number of potential explanations. Firstly, episodes of chlamydial infection in adults are known to be relatively short in duration, and therefore are more difficult to observe in cross-sectional and longitudinal studies without very frequent testing [29]. However, even with this study limitation, one might have anticipated more detectable infection episodes with biannual testing, if there is indeed an underlying association between scarring progression and chlamydial infection in these cohorts. Secondly, the detection of infection (if present) in adults with scarring might be more difficult due to lower bacterial loads [30]. An alternative explanation is that other factors also contribute to the progression of established scarring in adults. This is consistent with studies from regions such as The Gambia, which has been known to have very low levels of active trachoma and chlamydial infection for many years, and yet at the population level incident trichiasis remains a problem [5,31].

A number of cross-sectional studies have investigated the relationship between scarring trachoma and non-chlamydial bacterial infection. These have generally found an association between increasing severity of cicatricial disease (trichiasis or conjunctival scarring) and positive non-chlamydial bacterial cultures and between the presence of clinical inflammation and a positive non-chlamydial bacterial culture [8,32–34]. It is possible that these bacteria could continue to drive on-going innate pro-inflammatory responses in the conjunctiva. Another possibility is that prior recurrent *C*. *trachomatis* infection modifies the conjunctival tissue, perhaps leading to imprinted epigenetic changes, similar to those observed in LPS tolerised macrophages or BCG exposed human monocytes, leading to modification of pro-inflammatory responses [35–37]. This would imply that stimuli from other bacteria could then stimulate pre-programmed chlamydia-like inflammation and pro-fibrotic pathways.

In this study we identified consistent associations between the presence of clinically apparent conjunctival inflammation and the increased expression of several factors: *S100A7*, *IL1B*, *IL17A*, *CXCL5*, *CTGF*, *CEACAM5* and *CD83*. Interestingly, the expression of *SPARCL1* was markedly reduced in the conjunctiva of individuals with clinical inflammation. We did not, however, identify consistent associations between progressive scarring and the expression of the genes measured. In the Tanzanian cohort there was an association between progressive scarring and borderline increased expression of *S100A7* and *IL1B*, however, this was not found in the Ethiopian cohort. In view of the association between clinical inflammation and the progression of scarring, it is possible that factors associated with inflammation also contribute to progressive scarring.

We have previously reported cross-sectional case-control studies of conjunctival gene expression in children with active trachoma and adults with scarring disease, using whole transcriptome microarray analysis and similar quantitative PCR techniques [10,12,27,38]. In these we found the pro-inflammatory cytokines/chemokines *IL1B*, *IL17A*, *CXCL5* and *S100A7* to be among those showing the largest degree of increased expression [12,38]. Moreover, in a two-year longitudinal study of individuals who had undergone trichiasis surgery, using a similar study design, we found that clinical inflammation was associated with increased expression of *S100A7*, *IL1B*, and *CXCL5* and recurrent trichiasis was associated with increased *S100A7* expression [39]. These responses appear consistent across different stages of disease. Expression of *S100A7*, *IL1B*, and *CXCL5* has been demonstrated in epithelial cells as part of the innate immune response to several bacterial pathogens [27,40,41]. These proteins are important components in the cascade of inflammatory responses, particularly neutrophil chemotaxis and activation.

The carcinoembryonic antigen CEACAM5 is part of the immunoglobulin superfamily, which is perhaps best known as a tumour marker. CEACAM5 is a cell surface receptor found on epithelial cells, and its expression may be enhanced by Smad3-mediated TGFβ signalling [42]. In addition to being involved in tissue regulation processes through cell-cell interactions, CEACAM5 is an adhesion molecule to which a variety of bacteria bind [43]. This may facilitate the colonisation of epithelial surfaces and there is some evidence that this might also suppress the local innate immune response through interfering with toll-like receptor (TLR) signalling.

The expression of the pro-fibrotic factor *CTGF* was also associated with clinical inflammation and may represent an important link between the inflammatory response and the development of scarring. We have previously found *CTGF* expression to be increased in the conjunctiva of children with active trachoma [27]. CTGF is important in a number of scarring disease processes in which it is constitutively over expressed. Its induction and activity are closely associated with and promoted by TGFβ [44–46]. It has matricellular protein functions, binding both to extracellular matrix (ECM) components and cells; it also promotes the production of several types of collagen [46,47]. Murine models suggest that CTGF may be required in addition to TGF-β for the development of permanent scar tissue.[48] *CTGF* expression may represent a useful biomarker for an active scarring process which warrants further study in trachoma [49].

The gene with the largest degree of reduced expression in our previous microarray experiment of scarring cases and controls from Ethiopia was *SPARCL1* (also known as Hevin) [12]. This observation was replicated in a second comparable study in Tanzanian adults.[10] SPARCL1 is another matricellular protein with important functions in regulating cell-extracellular matrix interactions (antagonising cell adhesion) and the production of ECM components [47]. In experimental models of corneal wound healing in SPARCL1 null mice, the lack of this factor was associated with increased fibrosis, excessive and irregular collagen, increased MMP activity and inflammation [50].

We expected this study design to identify host factors, such as conjunctival gene expression levels or patterns, that were associated with progressive scarring. Discovery of such factors would be useful biomarkers for the identification of individuals at risk of progression. However we have been unable to clearly identify single factors or combinations of factors from this set of genes to discriminate scarring progression. This would suggest either that they may not be involved in progression and a wider set of genes encompassing a more complete regulatory network needs to be studied, or that transcript levels are not representative or of sufficient magnitude to give discriminatory power. It is also possible that for this complex disease in which multiple genes each exert minor effect critical pathways need to be studied rather than single factors.

This study has a number of limitations. As discussed above, biannual sampling may not be sufficiently frequent to detect an association between *C*. *trachomatis* and scarring progression, as infection events in these cohorts were very rare. We found that the comparison of high quality photographs was a straightforward method to assess progression. However, it may be easier to detect progression when there is less scarring at the outset. Overall, there was a trend to less inflammation towards to the end of the study. This could be attributable to general secular trends or the introduction of the epilation intervention in the case of the Ethiopian cohort. However, this undermines the overall conclusion that conjunctival inflammation was associated with scarring progression. The measurement of gene expression in conjunctival surface swab samples offers a non-invasive and repeatable method of assessing the immuno-fibrogenic response. However, many regulatory networks may be independent of transcriptional regulation and expression may not always reflect the level of functional protein. The conjunctival swab sample may also be less informative about events taking place below the epithelial surface.

In summary, we found evidence of progressive scarring in cohorts of individuals from Ethiopia and Tanzania with established conjunctival scarring. In both populations the progression was associated with conjunctival inflammation but not detectable *C*. *trachomatis* infection. The drivers of this late stage disease remain unclear. Several pro-inflammatory cytokines and matricellular proteins were associated with conjunctival inflammation, which in turn is associated with progressive scarring. Matricellular proteins such as SPARCL1 and CTGF have received little attention in trachoma to date; it is plausible that they have an important role in the development of scar tissue. However, we did not find a consistent association between the expression of specific factors and progression of scarring, although in the Tanzanian cohort there is some evidence that *IL1B* and *S100A7* expression are increased. Trachoma control programmes need to anticipate progressive scarring disease in previously endemic populations by maintaining surveillance for trichiasis and surgical services possibly for many years after chlamydial infection has been controlled.

## Supporting Information

S1 Checklist STROBE checklist. (DOCX)

S1 Table Tarsal conjunctival scarring grading system. (DOCX)
