# Supplementary material for: Pathogenesis of Progressive Scarring Trachoma in Ethiopia and Tanzania and Its Implications for Disease Control: Two Cohort Studies
Source: PLoS Negl Trop Dis. 2015 May 13;9(5):e0003763. doi: 10.1371/journal.pntd.0003763 (PMC4430253; doi:10.1371/journal.pntd.0003763)
Supplement: S1 Table — (DOCX) [file pntd.0003763.s002.docx]

| **Grade** | | | **Definition** |
| --- | --- | --- | --- |
| S1 | |  | Scarring occupying <⅓ of the upper lid: |
|  | S1a | | One or more pinpoint scars and/or a single line of scarring less than 2mm in length |
|  | S1b | | Multiples lines of scarring less than 2mm in length |
|  | S1c | | One or more lines/patches of scarring each 2mm or more in length/maximal dimension |
| S2 | |  | Patches of scarring occupying in surface area ≥ ⅓ but < ⅔ of the upper lid |
| S3 | |  | Patches of scarring occupying in surface area ≥ ⅔ of the upper lid |
